# Externalities, scarcity, and abundance

**DOI:** 10.3389/frma.2022.1111446

**Published:** 2023-01-11

**Authors:** Brett M. Frischmann, Giovanni B. Ramello

**Affiliations:** ^1^Department of Business and Economics, Villanova University, Villanova, PA, United States; ^2^Department of Economics and Statistics, University of Turin, Turin, Italy

**Keywords:** externalities, institutions, abundancy, scarcity, transaction costs, comparative institutional analysis

## Abstract

**Introduction:**

Do externalities work and matter differently in a world of scarcity vs. a world of abundance? In this article, we critically examine the economic phenomena of externalities. The concept of externality, an important idea in economics and law, is useful in exploring the complex and dynamic relationships between resource supply and human flourishing within various sociotechnical systems.

**Methods:**

First, we define the basic concept and explain why it is fundamental to economic analysis of complex social environments Second, we briefly survey the intellectual history of externalities with the goal of tying together a few different strands of economic theory and providing a roadmap for a general theory of externalities. This discussion highlights a latent conflict between those who pursue and those who resist perfectibility (optimization) of social systems by internalizing externalities. Third, we compare externalities in worlds of scarcity and abundance.

**Results:**

This article develops the theoretical framework, including a brief intellectual history and notes toward the development of a general theory of externalities. As a conceptual tool, externalities enable one to identify and examine social interdependencies and to map their causes and consequences. Externalities provide evidence of social demand for governance institutions. This descriptive utility can and should inform normative analysis, the design of governance institutions, and comparative institutional analysis. We also raise a series of (mostly empirical) questions that should frame comparative institutional analysis and evaluation of different externalities in the digital networked world.

**Discussion:**

We focus on the scarcity and abundance of knowledge resources and the (technological) means for participating in the production, dissemination, and modification of such resources. In the real, necessarily imperfect world where abundance and scarcity vary across resources, people, and contexts, externalities persist, indicate social demand for governance, and inform comparative analysis and design of governance institutions.

**JEL classification:**

D62, B52, D02.

## 1. Introduction

Do externalities work and matter differently in a world of scarcity vs. a world of abundance? Over the past decade, many prominent scholars and thought leaders have argued (hypothesized) that increasing abundance of various types of knowledge resources and the technological means for participating in the production, dissemination, and modification of such resources will lead to substantial impacts, changes, and even disruptive transformation of existing political, economic, and social systems. This article does not empirically test this rather broad claim. Instead, we presume there is some inevitable truth to the generic claim, which anyone living in the twenty-first century can appreciate, and focus more directly on understanding the mechanisms, namely, in how scarcity and abundance of knowledge resources shapes political, economic, and social systems and, as we shall see, vice versa. This inquiry forces us to interrogate the conventional economics of externalities.

The concept of externality, an important idea in economics and law, is useful in exploring the complex and dynamic relationships between resource supply and human flourishing within various sociotechnical systems. Unfortunately, the externality concept is easily confused in making prescriptions. For example, economists often consider externalities to be a prime example of market failure (Papandreou, 1994). As we explain below, this is a bad heuristic. In reality: Externalities are sometimes evidence of market failure and other times evidence of market success. Furthermore, externalities sometimes are not primarily about markets failing (or succeeding) but instead concern political or other social systems failing (or succeeding) (Claassen, 2016). Not surprisingly, a bad heuristic can lead to bad prescriptions to remedy supposed failures.

Before we examine how externalities work and matter in worlds with varying degrees of scarcity and abundance, we provide a series of clarifications to help avoid the problems that plague conventional theories. We explain that externalities (i) are system-independent, (ii) always concern the interdependent and functional relationships between people and environments (resources, both natural and built), (iii) vary according to the set of values people have, and (iv) often, though not always, give rise to social demand for governance. As institutional economists recognize, externalities and institutions are inexorably intertwined. Yet governance institutions, which are by no means limited in focus to internalizing externalities, are themselves socially constructed resources that comprise and shape the built environments within which people live and develop their beliefs, preferences, and capabilities. This unavoidable fact adds a layer of complexity to the analysis that we do not fully describe in this paper and thus leave for other work.^1^ But we mention it because it is relevant to understanding how externalities and corresponding governance institutions work and matter differently in a world of scarcity vs. a world of abundance.

The real world is necessarily imperfect. It is complex and messy. Scarcity cannot be eliminated, and thus, a “world without scarcity” will never exist and can only be theorized. Nonetheless, which resources are scarce and to what degree does change over time and is a critical issue. Economics generally acknowledges these facts. In Part 2, we discuss the economics of externalities with these facts and the interdisciplinary audience of this journal in mind.^2^ In Part 3, we engage the hypothesis noted above regarding abundance. First, we briefly consider the abstract idea of a world of absolute abundance (without scarcity) and note how Ronald Coase used and others have abused this idea. This discussion situates our analysis in the broader themes of the Special Issue of Frontiers. Second, we consider the more realistic idea of a world in which specific sets of knowledge resources are increasingly abundant. Such a world can exist, and in such a world, externalities matter.

In our modern digital networked world, externalities are, in fact, ubiquitous. We hypothesize that there are more externalities than ever in human history, social interdependence is at an all-time high, and social demand for governance is unmet and on the rise. Wishful thinking and appeals to abundance-enabled innovation, disruption, and democratization too easily distract, dissemble, and ultimately, disable comparative analysis and design of appropriate governance institutions. Accordingly, in Part 3, we offer a series of (mostly empirical) questions that challenge such appeals and frame interdisciplinary research needed to support comparative institutional analysis.^3^

## 2. Externalities in a world of scarcity

### 2.1. Definition and an abbreviated intellectual history

Most economists would agree on a rather standard and common definition of externalities that can be put in the following terms:

*Externalities are benefits or costs realized by one human being as a consequence of another human being's activity without a full accounting of the effects by the parties*.^4^

Based on this definition, one can say that externalities are rather familiar. We generate and realize externalities daily by virtue of our experiences in an interdependent society.^5^ Consider how many of your actions have small but nonetheless real effects on others around you. Many effects are small in magnitude and seem trivial—say, the effects of one person's loud cackling laugh on others trying to read at a coffeehouse. Such effects may add up and become more significant if persistent or widespread—if the cackler persists for a long time, perhaps every morning... or consider a person chatting loudly on her cell phone every morning on the public transit bus... or a person that maintains a beautiful flower garden to the benefit of those who pass by on the way to the bus... and so on. Textbook examples are legion. Negative and positive externalities are ubiquitous (Laffont, 2008).

Despite such familiarity and general agreement on the basic definition, the meaning and relevance of externalities has been contested in economics for many years. Acknowledging that “externality is an ambiguous concept,” Harold Demsetz suggested that “every cost and benefit associated with social interdependencies is a *potential* externality” (Demsetz, 1967, p. 348 [italics added]). In his view, externalities exist only where benefits or costs are not taken into account by parties because “the cost of transaction in the rights between the parties (internalization)... exceed[s] the gains from internalization.” In a similar vein as Demsetz, Kenneth Arrow insisted that the existence or non-existence of externalities is a function of the relevant institutional setting, incentive structure, information, and other constraints on the decision-making and exchange possibilities of relevant actors (Arrow, 1970; see also Papandreou, 1994, p. 13–68). Arrow connected externalities to the absence of a functioning market (Arrow, 1970, p. 59–67), essentially equating an externality with an incomplete or altogether missing market (Cornes and Sandler, 1996, p. 40–43).

In mainstream economics, externalities are one possible cause and even represent one form of market failure (Bator, 1957; Laffont, 2008). Externalities are one reason given in most economics textbooks to explain how markets fail to allocate resources efficiently. This has been standard, at least since Paul Samuelson's seminal work on public goods (1954).

The perceived problem is that externalities are not fully factored into a person's decision about whether, how, and how intensely to engage in an activity, and consequently—that is, as a result of the incomplete consideration, externalities may have a distorting effect on market coordination and allocation of resources. The linking of externalities to market failure suggests the following hypothesis:

H1: *Too few (many) resources will be allocated to activities that generate positive (negative) externalities because persons deciding whether and how to allocate resources to such activities will fail to account for the full range of benefits (costs)*.

And the following (counterfactual) hypothesis:

H2: *If the unaccounted-for benefits (costs) were taken into account, or internalized, the actors would behave differently, reallocating their resources in a more efficient manner*.

Distortions manifest both on the supply side, in terms of reduced incentives to invest in what would otherwise be optimal supply, and on the demand side, in terms of lost signals about what consumers want and where investments should be directed (Laffont, 2008). The “lost signals” description follows from the Arrow's notion of externalities as missing markets or unpriced exchanges.

We can describe the supposed market failure at two different levels of abstraction. First, at a micro level (partial equilibrium, see, e.g., Buchanan and Stubblebine, 1962), it may be seen as the consequence of an imperfection in the market for some specific good or service generated by or otherwise attributable to a specific activity. One prominent example involves public goods (Samuelson, 1954).

Consider an example: *silence on public transportation*. Silence (noise) can be quite valuable (costly), is non-rivalrously consumed, yet is often underproduced (overproduced). Individual producers contribute without fully capturing or accounting for the benefits (costs) realized by others; there typically is no market exchange. The shared environment of public transportation is easily congestible, however, by one person or a few. While those who value jointly produced silence might coordinate with each other and even engage in an exchange with those who would break the silence, such transactions are far and few between. Social norms and other informal governance mechanisms may work in some contexts, but not in others.^6^ Legal rules might even be adopted. But at what cost? A comparative analysis of institutions available to solve this collective action problem can get quite complicated. The point here is simply that actors being quiet and noisy may generate externalities as their actions generate benefits and costs for others in their vicinity. Whether or not any given level of silence/noise is optimal is highly contextual and may be difficult to assess. In this case the very notion of optimality depends on a partial equilibrium analysis, which essentially means, pretending all other markets and non-markets work perfectly. Below, we discuss some shortcomings of this style of analysis (see also Frischmann, 2012, p. 53–57).

Second, at a macro level (e.g., general equilibrium, see Arrow, 1970; Papandreou, 1994), the supposed market failure may be seen as an imperfection in the *market for markets*. A market may be missing altogether (Arrow, 1970; Berta, 2017). There are many reasons why this might be the case. We discuss some below. The basic idea is that markets are themselves a complex public good that must be supplied by people. Markets themselves—through the activities of market participants—generate many different types of positive and negative externalities, and as recent research has examined, markets are often a form of knowledge commons (Frischmann et al., 2014, 2017; Dekker and Kuchar, 2021). And so, like other public and social goods, markets themselves may be underproduced.

Demsetz (2008) argued the market for markets is presumptively an efficient means for assessing when the benefits of internalization exceed the costs of internalization, and thus, markets, like property rights, will come into being when it is efficient to internalize externalities. Specifically, he said: “Just as the market dictates that there will be no good X if the cost of producing X exceeds what people are willing to pay for it, so the market dictates that there will be no market if the cost of producing the market exceeds what people are willing to pay for it” (Demsetz, 2008, p. 131).

Frischmann (2009) replied that this view mistakenly “equates supply and demand for property rights [and] other internalization mechanisms such as regulation... with a market.” Demsetz extended partial equilibrium assumptions to the market for markets, which is not justified since it only pushes the analysis up a level of abstraction and does not deal with the complex interdependencies of externalities flowing within and between markets and non-markets (or market and non-market systems). Frischmann (2009, p. 815 [italics added]) explained:

Participants in the *market for a market for X* are not likely the same (complete set) as the participants in the *market for X*, nor are the third parties affected by the actions of either set of market participants the same. We cannot assume that everyone participates in each market or in some macro-market-for-potential-markets without simply assuming away the notion of third-party effects altogether.

The market for markets frame presumes the market system is the default social system for social coordination and governance. In reality, political and other social systems play a (more) significant role in supplying governance institutions, including those necessary for markets.

The existence of silence (noise) on the train, for example, is not well explained by an economic analysis of whether a market exists and whether transaction costs for creating such a market are too high. Of course, one can contrive a model or tell a story about non-existent property rights and high transaction costs, but such analysis borders on tautological. Most people detect the handwaving and intuitively know that in most cases, a better explanation is rooted in social norms and cultural attitudes.^7^ Economic analysis has a lot to offer, especially comparative institutional analysis and economic sociology. But much more detail is needed than facts about transaction costs, property rights, and the (non)existence of a market.^8^ That a (luxury) market for silent travel, e.g., quiet cars on trains, may exist alongside other governance arrangements does not undermine the point.

Assuming (for now) the two hypotheses are true and externalities determine market failure, then how, according to conventional economic thinking, should society address the resource misallocation problem? For some time, most economists accepted Pigou's view that the government ought to “intervene” *via* the tax or regulatory system and force externality-producing agents to fully account for their actions (Pigou, 1932). Thus, producers of negative (positive) externalities, such as pollution (education), should be taxed (subsidized) at a level that aligns private and social costs (benefits) (Cornes and Sandler, 1996, p. 72–78).

Coase (1960) challenged to the “Pigovian tradition” and gave credence to property rights as an alternative to the Pigovian solutions of government taxation or regulation as a means of dealing with externalities (De Meza, 1998, p. 270–73). Coase first suggested that in a world without transaction costs, which he referred to as “costs of market transactions,” all that would be needed for the market to function properly are well-defined property rights.^9^ In such a world, regardless of how property rights are assigned, everyone who might be affected by use of the resource to which the property right applies would bargain and (re)allocate rights in a manner that maximizes social welfare (Coase, 1960, p. 15–19; De Meza, 1998, p. 270). Of course, this theorem, sometimes referred to as the Coase Theorem to Coase's dismay, only holds in a world without transactions costs, *which is not the world we live in* (Coase, 1960, 1988; Ellickson, 1989; De Meza, 1998; Ramello, 2011; Frischmann and Marciano, 2015).

Coase mainly intended to emphasize the importance of considering transaction costs when comparatively evaluating institutional solutions to perceived market failures (Frischmann and Marciano, 2015). Coase anticipated a role for government above and beyond defining and enforcing property rights, but he thought that role should be evaluated contextually with a full understanding of the reciprocal nature of interdependent relationships^10^ and without a reflexive invocation of externalities to justify government action (Coase, 1960, p. 18; De Meza, 1998, p. 275). Buchanan and Stubblebine (1962, p. 381) agreed: “There is not a prima facie case for intervention in all cases where an externality is observed to exist.”

Buchanan and Stubblebine (1962) introduced the idea of *relevance*, by which they divided externalities worthy of attention and internalization from those deemed irrelevant. An externality is relevant only if its removal *via* internalization is Pareto improving^11^; this does not mean that internalization is necessarily justified because an evaluation of whether or not to internalize would turn on the costs of internalization, which vary according to technology, institutional context, and other factors. The point is more basic. The relevance/irrelevance distinction depends, in Buchanan and Stubblebine's analysis, on whether net gains can be made from a hypothetical, costless “trade” between parties. Absent such gains, internalization is not worth considering for it would not matter to the generating actors' behavior or incentives.

Demsetz (1967) took a different approach and advanced a theory of property rights evolution where imperfectly defined property rights improve and evolve to meet societal demand for the internalization of externalities.^12^. By definition (within economics, at least), property rights can be perfectly defined only in a world without externalities. In such a world, the range of “sanctioned behavioral relations among economic agents in the use of valuable resources” is completely and unambiguously delineated (Libecap, 1994, p. 145; Demsetz, 1998). As Libecap (1994, p. 145) explains, “In the limit, if property rights are so well defined that private and social net benefits are equalized in economic decisions, benefits and costs will be entirely borne by the owner.”

By insisting on property rights and institutions, Coase, Buchanan and Stubblebine, and Demsetz all meant to emphasize the importance of institutional means (solutions) to deal with external effects. In the absence of transaction costs, Coase explained, there is no need for government intervention because individuals can bargain and devise solutions to deal with the interdependencies that exist between them.^13^ Similarly, Buchanan (1965) claimed that individuals could devise “clubs” that allow individuals to internalize externalities and produce (local) public goods. If property rights are correctly defined, then externalities would be internalized (dealt with in the club). Yet, transaction costs exist, and clubs cannot always be built. When they can, they are not always perfectly efficient. Not surprisingly, the real world is awash in imperfectly defined property rights and externalities (Demsetz, 1998, p. 144; Epstein, 2002, p. 520; Frischmann, 2004, p. 967; Frischmann and Lemley, 2007).

### 2.2. Toward a general theory of externalities

In this section, we question and aim to correct some oversimplifications in the conventional theories. We begin with the basic definition: *Externalities are benefits or costs realized by one human being as a consequence of another human being's activity without a full accounting of the effects by the parties*. The definition entails three parts, each of which merits brief reflection:

*Benefits/costs realized by a human being* encompasses genuine adjustments in a person's welfare, interpreted for our purposes broadly to include wellbeing, capabilities for human flourishing, and other conceptions of values. While economics tends to prefer working with welfare measured in specific ways, one can reasonably describe externalities in terms of many different conceptions of benefits and costs that include human capabilities.^14^

*As a consequence of another human being's activity* requires a causal, functional connection between actions and consequences. Actions occur and cause effects in and through shared environments, physical and otherwise. The causal relationship and environmental conditions matter.

*A full accounting of the effects by the parties* requires effects be factored into decision making about the activity that generates the effects *as if* the parties are one party or, put another way, *as if* the decision is mutual. It thus requires more than mere awareness of or even knowledge about the effects by one or more of the parties.

The existence of an externality signifies an incomplete accounting of effects. There are many potential reasons. Incomplete accounting may be due to a lack of awareness, appreciation, or understanding (hereinafter “knowledge”) of how one person's actions generate consequences for others. While we group awareness, appreciation, and understanding together under knowledge for expository convenience, there are subtle and important differences between these states of mind, how they contribute to an incomplete accounting, and the types of governance mechanisms (interventions) that might enable a complete accounting. For example, institutions focused on transparency and notice may provide awareness but fall short with respect to appreciation and understanding. Knowledge about the dynamic relationships between actions, mediating environments, and consequences for other people is sometimes in the realm of common sense—as in the case of a person speaking loudly on the public train—but other times may be much more complicated—for example, when one contributes to “anonymous crowding,” a type of congestion, on shared networks (Cornes and Sandler, 1996, p. 355; Arruñada, 2017; Frischmann et al., 2019, p. 222–23).

Yet even with the necessary knowledge of such complexities, incomplete accounting may exist due to a lack of mutual concern (hereinafter “mutuality”). Making decisions *as if* parties are in fact one party is at the core of what economists mean when they refer to internalization. Theorists of collective action might instead use the words coordination and cooperation. A simple Prisoners' Dilemma provides a decent illustration. Even if both players are fully informed about the payoff structure and the consequences of their individual decisions, the dominant strategy eschews mutuality. *Knowledge is not enough*. The accounting is incomplete because of the lack of mutual concern. Institutions can provide a means for escaping the dilemma.

Mutuality is often socially constructed. It generally, but not always, requires some form of governance. Mutuality—internalization, cooperation, coordination, incentive alignment—can be genuine; that is, it does not need to be an *as if* condition. It can exist by virtue of a contract or joint membership in a common enterprise, such as a club, partnership, or corporation. It also can exist because the parties are very closely related, for example, family members. In the scenarios first noted, different governance institutions may effectively join parties such that one party making a decision that has a consequence for another should account fully for the effects, provided the actor has sufficient knowledge to do so. But even in such scenarios, mutuality is not guaranteed or inevitable (which is why we used the words “can” and “may”).

As if scenarios are legion. As if mutuality exists, for example, when social norms induce genuine consideration of others, including strangers, before acting in a manner that might affect them. Similarly, strict liability rules effectively require actors to make decisions in this fashion. As if mutuality also arises when property rights and other legal rules provide mechanisms for affected parties to seek recourse from actors who cause them injuries. There are plenty of examples to tease out, but the basic point is made.

Externalities mean an incomplete accounting; internalization of externalities entails a full accounting. Both components—*knowledge* (awareness, appreciation, and understanding) and *mutuality* (actual or as if)—matter. Different institutions can be designed to support one or both components. When engaging in comparative institutional analysis and assessing demand for governance, one must consider both components (see also Arruñada, 2017).

Our basic correction to the conventional economic theory about externalities is to cast aside the *externality as market failure* framing and replace it with the following.

First, externalities are fundamentally a product of *human beings* (inter)acting with(in) *environments*. Human beings are actors/agents with various capabilities and characteristics. They have their own independent will (beliefs, preferences, values, and intentions) and social relationships, and they are necessarily situated and even embedded in complex environments that shape their development and interactions. Multiple, complex, overlapping, and interdependent resource systems constitute those environments—the natural environment is one type and socially constructed (built) environments are another.^15^ These basic facts about the framing matter because they provide the contextual details or parameters necessary for identifying and evaluating externalities: Relevant parameters for such analysis include, *inter alia*, actors, actions, causal relationships, consequences/effects, environmental mediating factors, and relationships.

Second, externalities are not exclusively a market phenomenon. Rather, externalities arise as relevant phenomena in all social systems, including but not limited to markets. In various social contexts, incomplete accounting can lead to third-party effects. Externalities serve an evidentiary function by indicating demand for governance, which might be supplied in various forms by participants in market, political, or other social systems.

Third, externalities are not failures *per se*. Counterintuitively, externalities can be and often are evidence of successful operation of social systems and therefore do not require any internalization. For example, markets regularly generate externalities that need not and should not be internalized. Knowledge production in markets is a prime example where spillovers are widespread and socially desirable (Frischmann, 2007; Frischmann and Lemley, 2007; Ramello, 2011). This is success. The same can be said about political, academic, and other social systems. Success or failure depends on the contextual details.

The two hypotheses (H1 and H2 above) are thus sometimes valid, depending on the context, the activities, resources, technologies, and governance institutions, among other things. The critical *empirical* question, then, is to figure out when the hypotheses hold because that indicates there is a social dilemma, demand for governance, and an opportunity for improvement by internalization.

Fourth, internalization is no panacea. Internalization of externalities can be a solution when there is a problem to solve, but it also can be a problem to avoid when the two hypotheses do not hold. Knowledge and innovation are particularly useful examples. It is not just that producing and sharing knowledge can generate endless ripple effects that are too costly to internalize; it's that the ripple effects are often precisely the point. In fact, even if cheap, internalization can cause distortions that undermine the generation of socially valuable ripple effects, including cumulative innovation and cascading spillovers (Arrow, 1962; Scotchmer, 1991; Frischmann, 2009). For example, if the inventor of the microscope captured the full social value of the invention, it would reduce the incentive for countless scientists to make innumerable discoveries that are in the aggregate far more valuable (Arrow, 1962; Lemley, 2005; Frischmann and Lemley, 2007 [collecting sources and historical examples]). More generally, for infrastructural public goods for which a significant fraction of surplus is attributable to productive (re)use, internalization may affirmatively reduce social welfare (Frischmann, 2012). Ultimately, the case for and against internalization depends on the context and the scope of the analysis.

One way to see the third and fourth points is to reconsider Buchanan and Stubblebine's analysis of relevance. Buchanan and Stubblebine suggest that an externality is relevant only if its removal *via* internalization is Pareto improving; otherwise, it is irrelevant and need not be considered. The assumption is that the parties would not transact because there are no gains, and so it must be deemed irrelevant. There is no social dilemma, no problem to solve; internalization is inefficient. But what if their joint actions generate external effects that make internalization Pareto improving and thus worthwhile, although not market accountable?

For example, suppose A makes noise whistling on the bus and disrupts B, who cannot concentrate while A whistles. B would be willing to pay $1 to A if A would stop. This is not enough, however. A enjoys whistling and would only be willing to stop and forego such enjoyment for $2. According to Buchanan and Stubblebine, since there is no gain to be made *via* transaction, the externality is irrelevant. It need not and should not be internalized, even if internalization were itself costless. But suppose B is a writer, and on the bus, she writes interesting threads on Twitter that hundreds of followers enjoy (~$0.02 per follower). Further suppose that some fraction of her followers shares the threads with their followers, and that some fraction also adds their own content to the threads. We could go on extending the scope, the types of activities, public goods produced and shared, and interdependencies. We could change the medium (social technology of interaction) too. The point is that (ir)relevance and the corresponding economic case for internalization for each externality (externality-generating activity) depend entirely on how many interdependent market and non-market interactions one incorporates into the analysis. No matter how much we extend the analysis to markets, we cannot capture all social interdependencies and associated dynamics unless we make society coincide entirely with the market.^16^ Partial equilibrium analysis may be useful in making things tractable and working up a model to examine specific interactions, but it can be dangerously myopic (Frischmann, 2013).

One might wonder whether this example proves too much. It would seem to apply to countless examples of externalities, such as environmental pollution that inhibited an author from writing. Our example is one of millions we could describe. Silence is a public good that is valuable to some meaningful degree because it affords people opportunities to be productive in certain ways including but by no means limited to writing.^17^ Frankly, a healthy physical environment (free of pollutants) similarly affords people opportunities to be productive in certain ways including but not limited to writing. How health impacts productivity matters. It is structural. The argument applies to countless examples of externalities. That is the point.

Finally, a fundamental shortcoming made when examining externalities is to couple partial equilibrium analysis (and associated assumptions) with prescriptions focused on the pursuit of optimality or the perfectibility (optimization) of social systems. To develop this argument, we return to Paul Samuelson's seminal work on public goods.

Samuelson (1954, p. 387) suggested that since public goods simultaneously enter the “indifference curves” or “consumption functions” of many people, optimal production would have to account for the aggregate value for the consuming population. Thus, investment in production of a public good should expand so long as the aggregate marginal benefit to consumers exceeds the marginal cost. The optimality condition is framed in terms of marginal rates of transformation and substitutions as follows: Public goods production should expand until the marginal rate of transformation equals the sum of the marginal rates of substitution.

Accurately measuring demand and achieving optimality are difficult because consumers may act strategically and understate their actual preferences hoping that others will bear a greater proportion of the costs. This is known as the preference revelation problem. Competitive markets struggle with measuring demand for public goods, and while government could solve the demand revelation problem in some contexts through voting and political processes rather than market processes, Samuelson recognized that all of these processes are imperfect and thus optimal production would be elusive.^18^

The Samuelson condition indicates whether public or private investment in public goods production is justified, and effectively that evaluation is situated at the margin between investment in further public goods production and alternative investment opportunities (e.g., in private goods production). Here is what that means: Imagine you must evaluate a stream of potential investments. Specifically, you must decide whether to expand investment in public goods production. Expanding investment might mean investing more in an existing public good to improve its quality or investing in a new public good. Either way, the point is the same. For each potential public good investment, one must compare the aggregate benefits to the production cost, which includes the cost of capital and opportunity costs associated with alternative investment opportunities (i.e., rate of substitution).

In the basic model discussed thus far, the basis for measuring benefits to be aggregated is consumer preferences or willingness to pay for the public good in question. This model effectively assumes a single market, the public good market. Even if we assume consumers do not actively conceal their true preferences in a deliberate effort to free ride, demand measurement problems may persist, and optimal production may remain practically impossible.

The demand side analysis gets quite complicated when the public good is used productively, rather than merely consumed, and such productive use itself generates externalities.^19^ Recall our bus-riding author who used quiet/silence (public good 1) productively to produce Twitter threads (public goods 2, 3, … n), and followers who then shared those goods and by adding their own comments produced others (public goods n+1, n+2 …). Even if consumers cooperate and accurately reveal their preferences for some of those public goods, those preferences do not account for various third-party and structural effects. Unless externalities are internalized throughout the entire system (incomplete markets are completed, missing markets are made functional, etc.), which is impossible in the real-world, we must acknowledge and grapple with systematic demand side problems of both types—distortions associated with measuring actual consumer preferences and distortions associated with externalities (Again, there is nothing special about this example. We could describe countless other familiar examples with the same basic structure.).

The demand measurement problems posed by measuring actual consumer preferences and significant cascading external effects call into question the utility of marginal analysis and focusing on optimality conditions. Samuelson anticipated this point in an essay reflecting on his public goods theory:

Having called attention to the nature of the [first demand measurement] difficulty, I do not wish to be too pessimistic. After all, the world's work does somehow get done. And to say that market mechanisms are non-optimal, and that there are difficulties with most political decision processes, does not imply that we can never find new mechanisms of a better sort (Samuelson, 1958, p. 334).

[It] should be possible for the theorist to go beyond the polar cases of (1) pure private goods and (2) pure public goods to (3) some kind of a mixed model which takes account of all external, indirect, joint-consumption effects. I shall not write down such a mathematical model. But if I did do so, would we not find—*as Pigou and Sidgwick so long ago warned us is true of all external economies and diseconomies -that the social optimum could not be achieved without somebody's taking into account all direct and indirect utilities and costs in all social decisions?* (Samuelson, 1958, p. 335; emphasis added).

Now some may read this passage and believe Samuelson was making the case for a centralized decision maker such as the government. But this seems a stretch. Samuelson recognized the importance of external effects and the severe limits they posed on efforts to perfect both market and government systems and thus to achieve optimal production of public goods. Recognizing those limits, he suggests a continued search for “new mechanisms” might be worthwhile. His reference to Pigou and Sidgwick and “somebody's taking into account all [effects] in all social decisions” implicitly acknowledges that the fundamental limit is a full accounting, which as we explain above, entails *both* knowledge (awareness, appreciation, and understanding) and mutuality.

We live in a very complex second-best world evidenced by the prevalence and variety of external effects (Lipsey and Lancaster, 1956). Attempts to perfect one market should be expected to cause unpredictable and often harmful distortions in many other markets and non-markets. Those who are optimistic about the perfectibility of social systems, including markets, may believe that abundant data and powerful computational technologies will reduce complexity, eliminate externalities, and enable optimization across markets and non-markets. However, there is no empirical support for such beliefs. To the contrary, social interdependencies multiply, complexity increases, and externalities abound (We discuss this claim further below in the context of the Internet, digital networked technologies, and abundant knowledge).

*Pursuing optimality in this case is quixotic*. We should set aside optimality conditions and instead focus on how to improve market, government, and other social systems (and even new mechanisms) for the bulk of investments that are not at the “edge” in terms of being the last marginal projects that would satisfy the Samuelson conditions. We simply know too little about the territory leading up to the edge. To make the analysis tractable, we have to assume away (and thus ignore) too much.

Speech is a useful example. Speech is a communicative activity that regularly generates externalities, both positive and negative. Speech generally entails the sharing of public goods (ideas, facts, stories, rumors, falsehoods, knowledge, etc.), and such sharing often has direct and indirect effects. Speech affects social interdependence in many ways. Not surprisingly, we do not aim to optimally produce speech. It makes little sense to rely on governments or markets to optimally produce speech. It is simply too difficult to even begin measuring demand, and not just because some consumers will misrepresent their preferences in the hope of free riding. The knowledge requirements alone are hard to fathom, and mutuality is, in many cases, impossible. There are too many complex interdependencies. Internalization is not the overriding social objective, and while a relevant consideration, transaction costs are not sufficient explanation. Speech externalities are expected and encouraged. Indeed, abundant speech externalities are one of the foundational elements of a democratic society, especially one committed to pluralism.

## 3. Scarcity, abundance, and externalities

Recall the motivating hypothesis noted in the Introduction (and drawn from the themes of the conference and special issue) that increasing abundance of various types of knowledge resources and the technological means for participating in the production, dissemination and modification of such resources will lead to substantial impacts, changes, and possibly even disruptive transformation of existing political, economic, and social systems. Our (modest) claim is that, properly understood, the concept of externalities remains useful in exploring the complex and dynamic relationships between resource supply and human flourishing within various sociotechnical systems.

In previous sections, we described externalities as economic but also social phenomena. We had the real world in mind, and that means, we have been talking about how externalities work and matter in a world of scarcity. We now turn our attention to the question of how externalities work and matter in a *world without scarcity*, to use the phrase suggested in Mark Lemley's provocative 2015 article, *IP in a World Without Scarcity*.

There are a few ways to understand the world without scarcity.^20^ We discuss three.

First, we imagine a *world without scarcity*, which we could also call a world of absolute abundance. Now this is easy to say but hard to describe. What would it mean for all resources to be abundant? It could mean that all resources are (somehow) freely and limitlessly available. We might begin to venture into science fiction in trying to figure out how to describe such conditions, but we need not go that route. In economics, scarcity and abundance are a function of supply and demand. So long as supply well exceeds demand, scarcity may not be a relevant concern. But short of imagining a world with a very small population relative to available resources (cf. Hardin, 1968) or a population with very small demands (Frischmann and Selinger, 2018 [describing a world in which billions of people are made maximally happy at low cost by engineering their preferences]), it is difficult to take seriously the idea of a world without scarcity. One way or another, environmental resources, raw materials, attention, time, and many other resources will remain finite, in demand, and thus scarce.

Second, we revisit the Coasean world of zero transaction costs and perfect information. This is not a world without scarcity, but it is another idealized world. We mention it here because many of the flaws in law and economic reasoning based on the supposed Coase Theorem could reappear in this context. Notably, the Coase Theorem was Stigler's invention (Stigler, 1966, p. 113), not Coase's (Frischmann and Marciano, 2015). Generations of law and economics scholars have invoked the Coase Theorem and the ideal of a world without transaction costs to set baselines in theoretical models and frame prescriptive arguments about property rights. But this line of (law and) economics analysis often misses Coase's fundamental point. As Frischmann and Marciano (2015, p. 348–349) explain:

Coase had little faith in the toy model of a zero transaction cost world; he did not champion property rights or any particular social arrangement over any other. Rather, he critiqued partial analyses and emphasized that it is “desirable that the choice between different social arrangements for the solution of economic problems should be carried out in broader terms [than the value of production as measured by the market] and that the total effect of these arrangements in all spheres of life should be taken into account”.

Zero transaction costs, like zero scarcity, is an analytical red herring. A better, more realistic economic analysis must acknowledge the prevalence and importance of transaction costs and scarcity and focus on comparative institutional analysis.

Third, and more in line with the motivating hypothesis, we focus on specific resources and evaluate what it means for them to become more abundant. Once we abandon utopian dreams of ideal worlds and embrace reality, we must recognize that scarcity will remain relevant. The key economic questions concern which resources are scarce, which are abundant, and how do we govern their production, use, distribution, and so on. Of course, answering these questions necessarily requires careful consideration and evaluation of social interdependencies, which, as we have explained, are contingent upon the complex, dynamic relationships among people and their (resource) environments.

Thus, not surprisingly, externalities will remain and remain salient. The existence of externalities tells us different things, depending on the context. First, externalities might be evidence of failure or success of different social systems. This interpretation depends on the context and thus requires empirical testing of the two hypotheses (H1 and H2). Second, and related, externalities might manifest social demand for governance. There may be an opportunity to improve the state of affairs for those people who have interdependent relationships. Such an evaluation depends on their values and relationships and the effects of their actions. Third, externalities might indicate a lack of mutuality or relevant knowledge. This information would help in the design and comparative evaluation of institutions.

The motivating hypothesis about increasing abundance presses us to consider a series of questions about any externalities. In designing, comparing, and evaluating institutions to address governance challenges raised by externalities, we should ask:

- How are the externalities created?
- Which activities generate them?
- What economic, technological, social, and environmental conditions support these activities?
- What types of externalities are created?
- How are the externalities distributed to or realized by third parties?
- Do third parties realize costs and benefits cognitively with awareness and appreciation (and perhaps a willingness to pay if a market were to form), or are the costs and benefits realized more passively, taken for granted, or perhaps appreciated only vaguely?
- What are the relevant social systems? Do we need or want a market?
- Can we differentiate between types of externality-producing activities and types of externalities in a manner that is relevant to decision making despite problems with quantification and measurement?

These are representative questions; the list is by no means exhaustive. These are not arbitrary, however. The questions outline contextual details necessary for identifying different types of externalities and mapping parameters relevant to evaluation and institutional design (e.g., actors, actions, causal relationships, consequences/effects, environmental mediating factors, and relationships).

Returning to the motivating hypothesis, we might ask: What does increased abundance of knowledge resources mean for intellectual property laws that historically have been designed to create artificial scarcity and thereby facilitate markets? Lemley (2015) argued that the premises, purposes, and design of intellectual property laws needed to change in light of his predictions of increased abundance. He suggested that the Internet presaged 3D printing, Synthetic Biology and Bioprinting, and Robotics, that these technologies promised to eliminate scarcity (increase abundance) by enabling a much larger number of people—perhaps everyone—to access and use effective means of producing a wide range of intellectual and physical goods. Desai and Magliocca (2013) and Desai (2014) considered how digitization enabled decentralized production, lowered transaction and other costs, and disrupted existing business models and technological platforms. With a focus on 3D Printing, these scholars examined how markets and legal systems evolve in response to abundance, resolving some social dilemmas while creating others. Notably, Desai (2014) rejects the ideal of a world without scarcity, instead recognizing the scarcity will persist and continue to drive economic activities.

Another wave of technologies promising to destroy scarcity and generate abundance has emerged since 2015. We could discuss a range of supposedly smart tech or blockchain or NFTs or the metaverse or others. But it is not necessary to evaluate these or any other technologies that make grandiose promises about “democratizing” innovation, knowledge production, or other related activities (Marciano et al., 2020). Instead, we can make our point more simply if we focus on the Internet and consider why and how scarcity inevitably persists and what follows from that basic observation.

The Internet provides and shapes opportunities for individuals, firms, households, and other organizations to interact with each other and participate in various social systems. The scale and scope of possible and actual social interactions is staggering. To put it simply, a person can easily (with a click of button, at zero marginal cost) instantaneously communicate an idea to millions of people around the world. The idea can be about nearly anything. It can take various forms and be distributed in various media. It can generate positive and negative effects. It can be part of a continuous stream of interactions. And so on …

Everything that occurs on the Internet entails the communication of data between computers at the “ends” of interconnected networks. The bottom line, for our purposes, is that every interaction involving the Internet involves the generation and sharing of public goods (data), which are inputs into the production of public and social goods at the application, content, and social layers of the Internet ecosystem. Externalities are incredibly varied and ubiquitous (for details, see Laffont, 2008; Frischmann, 2012; Frischmann and Selinger, 2018).

In line with the motivating hypothesis, it is perfectly reasonable to assert the following: *Due to the Internet, more people have access to more data, knowledge, speech, and other intellectual resources as well as more means of producing and sharing such resources with others than ever before in human history*. These public good and infrastructural resources are increasingly abundant such that scarcity may seem nonexistent. But that is not really the case. Scarcity remains. In fact, scarcity of some resources has risen along with the abundance of others. Recall that scarcity and abundance depend on supply but also on demand. There may be an incredible, growing supply of intellectual public goods and infrastructural resources, but at what costs? On the supply side, inputs needed to produce and sustain such abundant supply may be scarce and increasingly so. Energy, time, and attention, for example, are rivalrous resources that for many suppliers (producers, curators, distributors, etc.) are increasingly scarce. On the demand side, what is the social demand for such resources? Do people want or need them? Do people access and use them? Again, at what costs?

That the Internet makes production and distribution incredibly easy and cheap—even costless—does not mean that consumption and productive use are costless. Counterintuitively, overabundance^21^ generates and exacerbates scarcity, as people must invest scarce resources (again, time, energy, and attention come to mind) to manage their affairs in a world drowned in data and digital networked technologies that mediate their lives and social interactions. Deciding what to consume, what to produce, what is worth paying attention, and even who to relate with and trust can be increasingly taxing endeavors in a world of abundance (Simon, 1971).^22^ One can only ignore these types of costs associated with consumption and productive use of abundant resources by donning partial equilibrium blinders and assuming away complementarities and interdependencies among abundant and scare resources.

This is a move we refuse to make. To be clear, we do not deny the initial descriptive claim that data, speech, and other intellectual resources as well as means of producing and sharing such resources are increasingly abundant. Rather, we insist on recognizing how scarcity of other complementary resources not only persists but likely increases because of increased abundance of data, speech, and other intellectual resources (C.f. Blevins, 2012).

This dynamic consideration raises others. For example, increased demand for and reliance on digital networked technologies to manage these costs of abundance may generate external effects on autonomy and other capabilities essential to human flourishing (Frischmann and Selinger, 2018). While it is beyond the scope of this paper to explore fully, we highlight, as a potentially fruitful area of future research, that the types of externalities, and corresponding social demand for governance, may shift from traditional welfare effects (more or less happiness, increased or reduced preference satisfaction) to capability effects (more or less capable, more or less autonomous, more or less rational, more or less creative, etc.). In evaluating the impacts of increased abundance on society, one might ask some basic questions. For example:

- *Are people more knowledgeable?*
- *Are people more capable of accessing and using the knowledge and knowledge-generating technologies in ways that improve their lives and the lives of others?*

The abundance of available data and knowledge does not mean that anyone knows everything or really anything at all. Despite wishful thinking of those who embrace the idea of cyborgian mergers of human minds with machines (Clark and Chalmers, 1998; Clark, 2003), abundant, Internet-accessible resources remain external to the human mind. Thus, to make the point crystal clear: Wikipedia is not part of anyone's mind. It is simply and quite incredibly an easily accessible source of abundant knowledge and means for producing and disseminating knowledge. There is no good reason to *presume* most people are capable of effectively accessing and using Wikipedia and many other abundant resources. Nor is there a good reason to *presume* that most people make the effort when they have reason to do so. The exciting fact of abundance too easily obfuscates empirical questions regarding what actual people can do and in fact do.

Some might dismiss our concern by suggesting that whether people avail themselves of abundant resources is simply a question of demand; unfortunately, such a perspective adopts a partial equilibrium, market-based frame and ignores structural conditions, failures in other markets, and non-market considerations. For example, Wikipedia may be accessible and quite useful to schoolchildren completing homework assignments. But technological conditions, such as lack of reliable Internet access, may be a structural barrier, and making effective use of Wikipedia and other abundant knowledge resources available online also may depend on digital literacy and other skills that are not taught or learned equally by everyone. Counterintuitively, the abundance of knowledge resources accessible by the Internet also might encourage forms of outsourcing, overconfidence, and reliance that undermine intellectual development and knowledge acquisition. Frischmann and Selinger (2018) explore various examples.

The bottom line is that there are many empirical questions that deserve attention if we are to say anything meaningful about how increased abundance affects society. It is important to investigate whether the abundant knowledge available on the Internet is, in fact, socially valuable. Broad claims about democratization or abundance do not provide *any* insight into quality or value. A more direct line of inquiry would focus on knowledge-based capabilities:

- *Are people more or less capable of solving problems?*
- *Are people more or less creative?*
- *Are people more or less literate, numerate, empathetic, etc.?*
- *Have the bounds of bounded rationality been stretched?*
- *Have people gained or lost common sense?*
- *Who has gained what intelligence?*

We can develop a long list of such questions regarding different types of human intelligence and capabilities (Frischmann and Selinger, 2018). Of course, these are generic and in practice entail a set of subsidiary questions that require interdisciplinary study. Nonetheless, we should consider these (and subsidiary) questions before jumping to any conclusions about what abundance means for society. If people are genuinely more capable in meaningful ways in their actual lives, then that would suggest many of the externalities from widespread participation in knowledge production and sharing on the Internet were in fact positive. However, if that is not the case, if people are demonstrably less capable in meaningful ways, then we should consider the possibility of negative externalities, looking to identify and study them, interrogating the mechanisms and causes, and evaluating social demand for governance. Of course, this is no easy task. As we explore below, the scale and scope of externalities is unprecedented and that only complicates the empirical work. The final question deliberately emphasizes distributional concerns in part to counter the “rising tides will lift all boats” style appeal of the abundance hypothesis and in part to prompt consideration of intelligence-based power, which by many (most) accounts in increasingly concentrated.

A related line of inquiry, suggested above, concerns the knowledge systems themselves and potential areas where abundance of some resources create or increase scarcity of others. For example, consider expertise, editorial skills, or other knowledge-related resources associated with quality intermediation (filtering, sorting, content moderation). Dramatic increases in quantity do not necessarily coincide with corresponding increases in quality. In fact, quite the opposite appears to be the case in many, though not all, sectors. Of course, to say this implies that there are accepted means for evaluating quality, which can be a contentious issue when relativism reigns and appeals to authority regularly are challenged. What is the relationship between (i) abundance of knowledge resources and (ii) concentration with respect to the tools, means, and human capabilities for evaluating the quality of such resources? Some might argue that along with abundant knowledge resources have come abundant tools for evaluating quality, ranging from decentralized forms of crowdsourcing to more centralized, platform-based forms of algorithmic content moderation. Others might criticize the availability and quality of these tools, their objective functions (e.g., how they evaluate, what they prioritize), and their impacts upon users and user capabilities (Frischmann and Selinger, 2018). It remains unclear whether abundant knowledge democratizes expertise and what that would even mean. Is expertise scarce, concentrated, or abundant? What about trust in experts, expertise, or expert systems? Again, we raise these questions to suggest that this line of inquiry deserves further scholarly attention if we are to evaluate what abundance means for society (Marciano et al., 2020).

In the imperfect world where abundance and scarcity vary across resources, people, and contexts, externalities persist, indicate social demand for governance, and should inform comparative analysis and design of governance institutions. The Internet example supports our argument. In our modern digital networked world, externalities are ubiquitous. We hypothesize that *there are more externalities than ever in human history* and *social interdependence is at an all-time high*. Recall how the Internet enables nearly instantaneous, incredibly low-cost production and distribution of public goods (data, speech, communications, even software applications). This has led to significant increases in the scale and scope of such goods produced and shared globally. The trillions (or more) of daily acts by ordinary people who produce and share such goods are an important reason for the basic motivating claim about abundance.^23^ Yet one can hardly imagine that many actors are aware of, much less appreciate and understand, the full range of effects that follow from their actions. Of course, people generally do understand some of the effects, the more immediate and direct ones as well as some indirect and attenuated ones. But in this context, what they know is necessarily only a fraction. We do not mean to imply anything about the signs or magnitudes of such effects, except that the magnitudes are not likely to be known by the actor. Of course, the signs and magnitudes of effects matter from a social perspective because they add up. Frischmann (2012) explained this in terms of social demand for the Internet and infrastructural applications-layer platforms. The overwhelming majority of actors may generate small-magnitude spillovers, but the net social impact from widespread production of small-magnitude spillovers can be massive. And at the same time and other extreme, a single actor may produce a “killer app” that generates incredibly large-magnitude spillovers, and the kick is that who will create it and what exactly it will be are impossible to predict ex ante—for both market and government actors. Back in 2012, Frischmann argued in favor of open infrastructures to support the full spectrum of spillovers, contending that the externalities were mostly positive and thus indicative of success rather than failure.^24^ Yet 6 years later, Frischmann and Selinger (2018) raised many of the critical concerns noted in the text above, questioning whether many of the external effects presumed to be positive were either negative or positive but accompanied by other complementary effects that were negative. These views highlight the persistence of externalities and the evolving social demand for governance.

Beyond *knowledge* about third-party effects, another obstacle to internalization in the digital networked world, and thus reason to believe that there are more externalities than ever before, is the *lack of mutuality online*. The Internet affords people around the world with the capacity to interact with a much larger number of weak ties and strangers than ever before in human history. Again, such interactions always involve the generation and exchange of public goods. While there is incredible variance in how people interact online and the degree to which such interactions generate externalities, our claim is that both genuine and as if mutuality are often absent, especially among strangers. While genuine mutuality would be difficult to imagine for strangers on the Internet, as if mutuality is possible with appropriate governance institutions in place, as demonstrated by some online communities and platforms that effectively govern shared resources and construct sustainable commons. In our view, widespread and substantial externalities among strangers online presents a strong indication of social demand for governance; design of appropriate governance institutions should account for both the knowledge and mutuality conditions necessary for internalization.

## 4. Conclusion

Motivated by the abundance hypothesis, this article revisited the economic phenomena of externalities. In the real, necessarily imperfect world where abundance and scarcity vary across resources, people, and contexts, externalities persist, indicate social demand for governance, and inform design and comparative analysis of governance institutions. This article developed the theoretical framework, including a brief intellectual history and notes toward the development of a general theory of externalities. It then explored a series of theoretical and empirical questions that challenge the abundance hypothesis.

## Data availability statement

The original contributions presented in the study are included in the article/supplementary material, further inquiries can be directed to the corresponding author.

## Author contributions

Both authors contributed to the article and approved the submitted version.

## Footnotes

^1^This remark resonates with the critique on the contractual and bilateral view on externality adopted by most of the law and economics analysis (Arruñada, 2017).

^2^We wrote this article for an interdisciplinary audience and conference, *Scarcity, Regulation, and the Abundance Society*, hosted at Stanford Law School on April 22–23, 2022, and organized by Professors Mark Lemley and Deven Desai.

^3^We recognize that some scholars find asking questions without also providing answers to be unscholarly or an insufficient contribution to knowledge. Our view, however, is that identifying gaps in knowledge and the series of questions that should help fill those gaps is an important contribution too often overlooked or dismissed by those who prefer asking and answering conveniently simple questions.

^4^We purposely excluded non-human species as well as human-built tools, such as corporations and software, from this definition.

^5^Market actors regularly generate externalities when making product and pricing decisions that affect other competitors. Economists have debated whether these effects are *really* externalities, whether the definition should be adjusted to exclude such effects, and whether a distinction should be drawn between technological and pecuniary externalities. See, e.g., Whitcomb (1972, p. 6) (equating externalities with technological externalities); Posner (2003, p. 7) (defining pecuniary externalities); Duffy (2005, p. 1081–85) (collecting sources and insisting that only technological externalities matter). Frischmann and Lemley (2007, p. 262–64) explain the technological/pecuniary distinction and why it does not hold “once we are willing to entertain the idea that the allocation of rights and thus wealth may have dynamic external effects.” We pick up on some of those arguments below. But the point here is simply to note that the basic definition in the text captures the general phenomenon, yet as the following paragraphs suggest, there remains confusion and disagreement about the relevance of different types of externalities to economic analysis.

^6^C.f. e.g., Kim (2012) shows why travelers on long distance bus travel (Greyhound Line buses) prefer silence as a strategy of disengagement from unknown others. But, of course, this does not apply always and everywhere.

^7^Of course, in such an explanation, property rights and transaction costs remain relevant factors that can influence and be influenced by social norms and cultural attitudes.

^8^We say more about those additional details below.

^9^While Coase (1960) did not explicitly use the term “property rights,” he referred repeatedly to legal rights concerning property.

^10^Coase critiqued the notion that polluter A causes homeowner B to suffer a negative pollution externality and viewed the harm realized by B as jointly produced by both A and B because they engage in interdependent activities—manufacturing and homeownership (Coase, 1960; Buchanan and Stubblebine, 1962, p. 381–82; Cornes and Sandler, 1996, p. 79–80, 86; De Meza, 1998, p. 273–74).

^11^Pareto superiority is the condition that determines voluntary exchange and makes markets useful. Otherwise, parties have no interest in using the market.

^12^Demsetz (2011, p. 655) later explained “[w]hereas Coase's work examined the consequences that followed from an existing private-ownership system, I sought to explain why such a system would come into existence.”

^13^As an anonymous reviewer noted, efficient bargaining in this scenario depends not only on zero transaction costs but also on an equal division of wealth.

^14^As we explore in more detail below, evaluating social consequences attributable to increased abundance of knowledge and digital networked technologies may require shifting focus from preference satisfaction and other conventional measures of welfare to human capabilities. Cf. Sen (1985, 1999, 2005) (capabilities); Hausman and McPherson (2009) (preference satisfaction).

^15^Many economists have struggled to differentiate externalities from the more general concept of interdependence. We do not. Externalities always, by definition, involve interdependence between two or more people. Such interdependencies are typically mediated through physical and social environments. Not all interdependencies are externalities, however. Most obviously, some interdependencies are fully accounted for by the parties. Perhaps less obvious are those interdependencies that do not involve consequences attributable to human activity.

^16^Granovetter (1985) expressed a similar criticism when discussing the embeddedness of economic activities within society. While a large fraction of human interaction may take place within markets, we cannot capture all the human interaction as a sum of markets.

^17^To preempt another potential objection, we acknowledge that while silence may be a public good with positive affordances for some, including writers, it also may be stifling for others who otherwise might produce different public goods, for example, by generating and sharing knowledge by speaking with each other. The complex tradeoffs only strengthen our argument.

^18^C.f. Samuelson (1954); Samuelson (1958, p. 334).

^19^Arrow (1962) made a similar observation in the case of knowledge, which is both an output and an input of inventive processes. Marchese et al. (2019) tries to give glimpses through a model of endogenous growth.

^20^We discuss all three because they surface in discussions of abundance. Lemley (2015), for example, posits and often refers to the “world without scarcity” (thus, evoking our first conception), but most of his analysis presumes scarcity persists for many resources (such as raw materials) and assumes abundance only for specific sets of knowledge resources (thus, evoking our third conception). Yet there are significant problems with alternating between the first and third conceptions, evoking one but relying on the other, and these problems may be seen through the lens of the use and abuse of the Coase Theorem (our second conception).

^21^The idea of overabundance in the sense of oversupplying knowledge may not resonate initially with an economist. Can there be too much of a good thing? What if the supply of such goods generates negative externalities akin to congestion externalities? This would require a congestible (potentially scarce) resource, such as conventional common pool resources. There are a few obvious candidates, such as attention and time. Other candidates include trust and expertise.

^22^“In an information-rich world, the wealth of information means a dearth of something else: a scarcity of whatever it is that information consumes. What information consumes is rather obvious: it consumes the attention of its recipients. Hence a wealth of information creates a poverty of attention.” (Simon, 1971, p. 40–42).

^23^We focus on ordinary users to make a point. Of course, we can extend the analysis to the incredibly wide range of professional, commercial, political, educational, governmental, scientific, and other organizational or institutional actors who also produce and distribute these types of public goods and only internalize a fraction of the externalities they generate.

^24^The argument is an applied version of the argument we made earlier about setting aside optimality conditions and instead focusing on how to improve market, government, and other social systems for the bulk of inframarginal investments.
